# A poor prognostic metastatic nongestational choriocarcinoma of the ovary: a case report and the literature review

**DOI:** 10.1186/s13048-021-00810-3

**Published:** 2021-04-22

**Authors:** Kimihiro Nishino, Eiko Yamamoto, Yoshiki Ikeda, Kaoru Niimi, Toshimichi Yamamoto, Hiroaki Kajiyama

**Affiliations:** 1 Department of Obstetrics and Gynecology, Nagoya University Graduate School of Medicine, 65 Tsurumai-cho, Showa-ku, 466-8550 Nagoya, Japan; 2 Department of Healthcare Administration, Nagoya University Graduate School of Medicine, Nagoya, Japan; 3 Department of Legal Medicine and Bioethics, Nagoya University Graduate School of Medicine, Nagoya, Japan

**Keywords:** Pure ovarian nongestational choriocarcinoma, Short tandem repeat analysis, BEP therapy

## Abstract

**Background:**

Pure ovarian choriocarcinoma can be gestational or nongestational in origin. Nongestational pure ovarian choriocarcinoma is extremely rare and the prognosis is thought to be worse than that of the gestational type in patients with metastatic disease. We present a case of metastatic pure ovarian choriocarcinoma with poor prognosis in which the origin was identified as nongestational by DNA short tandem repeat (STR) analysis.

**Case presentation:**

A nulliparous woman in her thirties with metastatic choriocarcinoma was referred to our hospital after initial treatment proved unsuccessful. Two months earlier, she had undergone brain tumor resection and histological examination confirmed choriocarcinoma. Serum human chorionic gonadotropin (hCG) concentration at initial diagnosis was 5030 IU/L. Two cycles of a combination chemotherapy regimen of methotrexate, etoposide, and actinomycin-D (MEA therapy), which is commonly used for gestational choriocarcinoma, was administered. However, the disease could not be controlled. Imaging modalities at presentation revealed tumor present in the left ovary and left lung, but not in the uterus, which led us think that the choriocarcinoma was nongestational. Bleomycin, etoposide, and cisplatin (BEP therapy) which is commonly used for nongestational choriocarcinoma (malignant germ cell tumor) and surgical resection of the uterus, bilateral ovaries, and an affected part of the left lung led to the nadir level of hCG, but the tumor relapsed and levels of hCG again increased. To investigate the origin of choriocarcinoma, we performed DNA STR analysis of tumor cells and oral mucosal cells. Analysis revealed the origin of the choriocarcinoma as nongestational, as the genotype of tumor cells entirely corresponded with that of oral mucosal cells. BEP therapy and chemotherapy regimens administered for nongestational choriocarcinoma and gestational choriocarcinoma proved ineffective, and the patient died 21 months after diagnosis of metastatic choriocarcinoma.

**Conclusion:**

Metastaic nongestational pure choriocarcinoma of ovary is an extremely rare and an aggressive disease, frequently resulting in poor outcome.

## Background

Pure ovarian choriocarcinoma can be classified into two groups based on origin: gestational or nongestational. The gestational type presents as either a metastasis of uterine choriocarcinoma, or as primary choriocarcinoma following ovarian pregnancy, including hydatidiform mole [1–3]. The nongestational type emerges as a malignant germ cell tumor, but is extremely rare because most malignant germ cell tumors are mixed-type and consist of various malignant components, including immature teratoma, dysgerminoma, yolk sac tumor, and choriocarcinoma [4–6]. Diagnosing pure ovarian choriocarcinoma as gestational or nongestational is essential, because the sensitivities to chemotherapy and prognosis of these two types appear to differ despite the identical pathological findings [7, 8]. DNA short tandem repeat (STR) analysis is a critical modality to differentiate the types independent of common clinical information, such as medical history and/or imaging findings. Herein, we report a case of metastatic primary pure ovarian choriocarcinoma identified as nongestational by DNA STR analysis that showed chemo-resistance to regimens administered for gestational choriocarcinoma as well as nongestational choriocarcinoma with a review of the literature.

## Case presentation

**Fig. 1:**
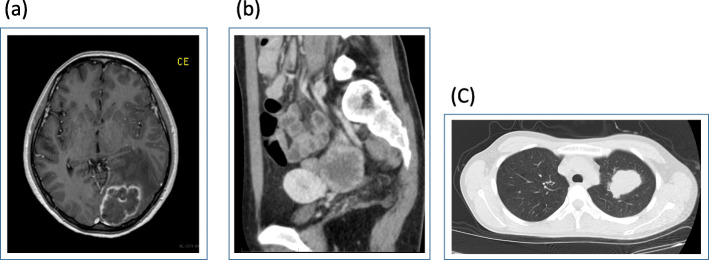
**a** Magnetic resonance imaging of the brain. Findings from contrast-enhanced T1-weighted magnetic resonance imaging of the brain. A tumor (4 cm in diameter) is seen causing midline shift in the left occipital lobe. The ridge of the tumor is enhanced. **b** Contrast-enhanced CT of the abdomen and pelvis. Contrast-enhanced CT of the abdomen and pelvis, showing a solid left ovarian tumor (5.5 cm in diameter) and enhanced with a ridge. **c** Chest CT Chest CT reveals metastatic choriocarcinoma in the left lung

A 38-year old nulliparous woman experienced headache, nausea, and a defect in the visual field over a period of days. Computed tomography (CT) and magnetic resonance imaging (MRI) of the head detected a tumor with midline shift in the left occipital lobe, 4 cm in diameter (Fig. 1a). As a result, she had undergone resection of the brain tumor. Histological examination of the resected specimen confirmed pure choriocarcinoma. Serum concentration of human chorionic gonadotropin (hCG) was 5030 IU/L. Serum carbohydrate antigen 125 and α-fetoprotein levels were within normal range. Abdominal and pelvic CT and MRI demonstrated a solid left ovarian tumor, 5.5 cm in diameter (Fig. 1b). However, no evidence of tumor in the uterus was seen. Chest CT revealed metastatic choriocarcinoma in the left lung (Fig. 1c). Consequently, her disease was diagnosed as primary pure ovarian choriocarcinoma and two cycles of a combination chemotherapy regimen comprising methotrexate, etoposide, and actinomycin-D (MEA therapy) which is commonly used for gestational choriocarcinoma, were administered. However, each administration of this regimen caused strong bone marrow suppression and a new metastasis appeared in the cerebellum after the second cycle of MEA therapy. Therefore, she was referred to our hospital.

**Fig. 2:**
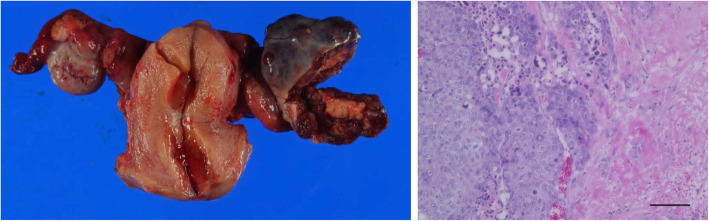
Macroscopic and microscopic findings. Macroscopic findings of the left ovarian tumor, showing massive central necrosis (left). Microscopic appearance of the tumor shows atypical cytotrophoblastic and syncytiotrophoblastic cells and necrosis (right). Original magnification, ´200; scale bar, 500 μm

**Fig. 3:**
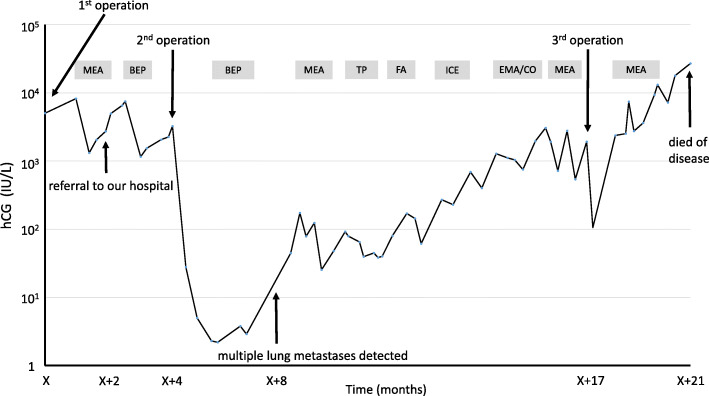
The trend of hCG. 1st operation: Brain tumor resection, 2nd operation: Total abdominal hysterectomy with bilateral salpingo-oophorectomy and segmentectomy of left lung, 3rd operation: segmentectomy of right lung, MEA: methotrexate, etoposide, and actinomycin-D, BEP: bleomycin, etoposide, and cisplatin, TP: paclitaxel and cisplatin, FA: fluorouracil and actinomycin-D, ICE: ifosfamide, cisplatin, and etoposide, and EMA/CO: etoposide, methotrexate, actinomycin-D, cyclophosphamide and vincristine

The third cycle of MEA therapy was administered with concomitant administration of pegfilgrastim to avoid bone marrow suppression. Gamma knife therapy was also performed for the cerebellar metastatic lesions. However, hCG levels increased continuously and the chemotherapy regimen was changed to a combination chemotherapy regimen of bleomycin, etoposide, and cisplatin (BEP therapy), which is commonly used for nongestational choriocarcinoma (malignant germ cell tumor). Two cycles of BEP therapy were effective and total abdominal hysterectomy was performed with bilateral salpingo-oophorectomy and segmentectomy of the left lung. Histologic examination of the left ovarian tumor demonstrated vast central necrosis and remnant marginal choriocarcinoma with no evidence of other germ cell elements, leading to a diagnosis of pure choriocarcinoma of the ovary (Fig. 2). BEP therapy and surgical resection achieved the nadir level of hCG (2.2 IU/L) (Fig. 3). However, two additional cycles of BEP therapy failed to achieve complete remission, and hCG level increased steeply when chest CT revealed multiple metastases in the lungs.

**Fig. 4:**
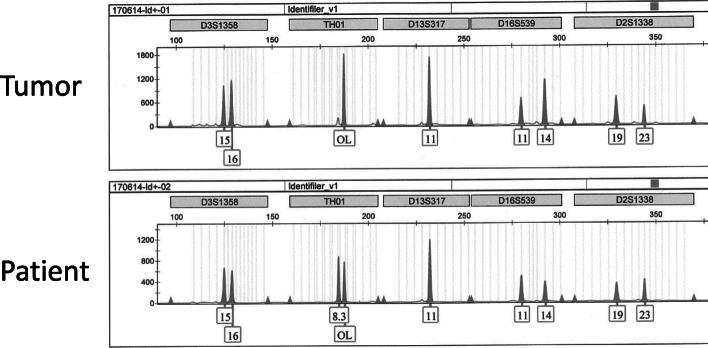
DNA STR analysis. DNA STR analysis reveals the genomic DNA alleles of the tumor are identical to those of the patient, exhibiting that the choriocarcinoma is nongestational

Her clinical history was reevaluated to identify the origin of choriocarcinoma and choose an appropriate chemotherapy regimen. She was unmarried, but had had sexual intercourse with her partner. Her menstrual cycle had been irregular since the time she had suffered subarachnoid hemorrhage due to rupture of an aneurysm on the posterior inferior cerebellar artery 10 months before development of the present illness. Therefore, the possibility of gestational choriocarcinoma subsequent to unrecognized pregnancy could not be ruled out. Consequently, we performed DNA STR analysis using a capillary electrophoresis system. Written informed consent was obtained from the patient for DNA extraction and DNA STR analysis, and this investigation was approved by the Ethics Committee of Nagoya University Graduate School of Medicine. DNA was extracted from oral mucosal cells of the patient and frozen cancer tissue, using a QIAamp® DNA Micro kit (Qiagen, Hilden, Germany) according to the protocol provided by the manufacturer. One microliter of extracted DNA was amplified using an AmpFℓSTR® Identifiler® Plus PCR Amplification Kit (Life Technologies, Waltham MA) for 15 STR loci and the amelogenin locus for gender determination, for a total of 16 DNA markers, as previously described [9, 10]. One microliter of amplified DNA was capillary electrophoresed on an Applied Biosystems 3130xl Genetic Analyzer (Life Technologies), and the detected peaks were genotyped automatically using GeneMapperID software version 3.2.1 (Life Technologies) as previously described [9, 10]. Part of the STR analysis is shown in Fig. 4. Genomic DNA alleles of the tumor were entirely identical to those of the patient, confirming that the choriocarcinoma was nongestational.

A combination chemotherapy regimen of MEA, paclitaxel and cisplatin (TP), fluorouracil and actinomycin-D (FA), ifosfamide, cisplatin, and etoposide (ICE), etoposide, methotrexate, actinomycin-D/cyclophosphamide and vincristine (EMA/CO) with segmentectomy of the right lung were administered after tumor relapse, but no chemotherapy regimens successfully suppressed tumor activity. The patient died 21 months after the diagnosis of choriocarcinoma.

## Discussion and conclusions

In this report, we have presented a case of metastatic pure ovarian nongestational choriocarcinoma that was differentiated from gestational choriocarcinoma by DNA STR analysis. This case showed resistance to chemotherapy regimens commonly administered for malignant germ cell tumor (nongestational choriocarcinoma) and for gestational choriocarcinoma.

When histological examination reveals pure ovarian choriocarcinoma lacking uterine lesions, as in this case, the nongestational is often presumed without careful attention, because gestational choriocarcinoma commonly originates from the uterus. Indeed, gestational choriocarcinoma without uterine lesions on initial presentation is very rare [11]. However, one paper reported a case of gestational choriocarcinoma (confirmed by DNA analysis) with renal and pulmonary metastases without any uterine lesions [12]. This may be due to spontaneous regression of the uterine tumor, possibly because gestational choriocarcinoma is a tissue allograft for the patient that induces strong immune reactions from the patient. In addition, not a few case reports have described primary ovarian gestational choriocarcinoma following ovarian pregnancy including hydatidiform mole [1–3]. In those clinical settings, uterine lesions are not necessarily present. Accordingly, precise differentiation of gestational and pure nongestational choriocarcinoma based on histological examination or lesion spread is impractical. A history of past pregnancies is thus crucial. However, even with such information, determining whether the choriocarcinoma is gestational or nongestational is difficult based on the pregnancy history alone, except in cases of patients who are sexually immature, have never had sexual intercourse, or are unable to conceive, because not a few spontaneous abortions pass unrecognized. In the present case, the patient had a fixed partner, had engaged in regular sexual intercourse, and had irregular menstruation cycles before the development of the disease, meaning that the possibility of gestational choriocarcinoma due to unrecognized abortions could not be ruled out.

A STR is a microsatellite consisting of a unit of 2–10 nucleotides repeated several to hundreds of times, and STR analysis allows evaluation of the highly polymorphic STR loci where each individual shows differing numbers of repeated sequences of nucleotides. DNA STR analysis is frequently used in forensic genetics [13] and population genetics [14] to match DNA profiles to an individual. Recently, STR analysis has been utilized to clarify the origin of choriocarcinoma and to differentiate gestational and pure nongestational choriocarcinoma when definitive evidence has not been forthcoming from pathological or other clinical findings [7, 15–20]. Because the genome in nongestational choriocarcinoma comprises only a maternal (patient) allele, whereas gestational choriocarcinoma contains a paternal allele, DNA STR analysis can precisely distinguish these two types of choriocarcinoma. In the present case, the tumor genotype entirely matched that of the patient, confirming the nongestational type.

Nongestational pure choriocarcinoma of ovary is an extremely rare, especially with metastatic disease (Table 1) [6, 21–26]. The prognosis of pure nongestational choriocarcinoma is widely considered to be worse than that of gestational choriocarcinoma despite these two types of choriocarcinoma being pathologically identical. If this is indeed the case, such differences may be because the genome of gestational choriocarcinoma is totally or partially different from that of the patient, whereas nongestational choriocarcinoma is genetically identical to the patient, leading to differences in host immunoreactions. However, given with rarity of these pathological entities, no reports have analyzed large numbers of choriocarcinoma cases and compared prognoses between types as diagnosed based on DNA analyses. Combination chemotherapy regimens employing methotrexate, etoposide, and actinomycin-D such as EMA/CO or MEA are administered as first-line therapies against gestational choriocarcinoma, and the complete response rate for these chemotherapy regimens against this disease exceeds 70 % [27]. Regimens including cisplatin such as TP and BEP therapies are applied for resistant or relapsed disease, with an overall survival rate with or without adjuvant radiotherapy or surgery of around 90 % [28]. Nongestational choriocarcinoma usually develops as a component of mixed-type germ cell tumor, and seems to show favorable prognosis following surgery and BEP regimen [29, 30]. When nongestational choriocarcinoma emerges as a pure type, however, the tumor behavior seems more aggressive. Cisplatin regimens are usually used for resistant or relapsed disease, as well as for gestational choriocarcinoma. In the present case, the tumor was confirmed as pure nongestational choriocarcinoma by DNA STR analysis, but showed multiple chemo-resistances against chemotherapy regimens typically administered against both nongestational or gestational choriocarcinoma.

**Table 1:** Reported cases of nongestational pure choriocarcinoma of the ovary with distant metastases at the time of diagnosis

Case	Authors (Ref)	Age	Side	Distant metastases	hCG (IU/L)	Surgery	Chemotherapy	Outcome	DNA analysis
1	Stevens *et al*., 1979 [21]	19	left	lung	80,000-160,000	TAH, BSO	MA, thiotepa	Died	No
2	Raju *et al*., 1985 [22]	16	right	lung	NS	not done	not done	Died	No
3	Tsujioka *et al*., 2003 [23]	19	left	lung	110,000	LSO, OM	EMA/CO	NED	Yes
4	Park *et al*., 2009 [24]	55	right	lung	64,838	TAH, BSO	BEP	NED	No
5	Gremeau *et al*., 2010 [25]	46	left	brain, lung	4,962	LSO	YES, details NS	Died	No
6	Exman *et al*., 2013 [26]	24	NS	lung	675,713	TAH, BSO, OM	BEP	NED	Yes
7	Rao *et al*., 2015 [27]	26	right	spleen, adrenal gland	8,160	RSO, OM, tumor resection of the spleen and adrenal gland	BEV	NED	No
8	Nishino *et al*., 2020 (present)	38	left	brain, lung	5,030	TAH, BSO, tumor resection of the lung	MEA, BEP, etc	Died	Yes

*NS *not stated, *TAH *Total abdominal hysterectomy,  *SO *salpingo-oophorectomy, *B *bilateral, *L *left, *R *right, *OM *omentectomy

*MA *methotrexate and actinomycin-D, *EMA/CO *etoposide, methotrexate, actinomycin-D, cyclophosphamide and vincristine

*BEP *bleomycin, etoposide, and cisplatin, *BEV *bleomycin, etoposide, and vincristine, *MEA *methotrexate, etoposide, and actinomycin-D

We have presented a case of metastatic pure nongestational choriocarcinoma that showed poor sensitivity to chemotherapy and the death of the patient. Precise differentiation of gestational and nongestational choriocarcinoma based on STR analysis independent of common clinical information such as the medical history and/or imaging findings is essential for determining appropriate chemotherapy regimens and evaluating the prognosis. With distant metastasis, pure ovarian nongestational choriocarcinoma is highly malignancy and the prognosis is poor.

## Data Availability

The datasets generated and/or analyzed during the current study are not publicly available due to individual privacy, but are available from the corresponding author on reasonable request.

## Abbreviations

STR: Short tandem repeat
CT: Computed tomography
MRI: Magnetic resonance imaging
hCG: Human chorionic gonadotropin
MEA: Methotrexate, etoposide, and actinomycin-D
BEP: Bleomycin, etoposide, and cisplatin
TP: Paclitaxel and cisplatin
FA: Fluorouracil and actinomycin-D
mini ICE: ifosfamide, cisplatin, and etoposide
EMA/CO: Etoposide, methotrexate, actinomycin-D/cyclophosphamide and vincristine
